# Adapting the James Lind Alliance priority setting process to better support patient participation: an example from cystic fibrosis

**DOI:** 10.1186/s40900-019-0159-x

**Published:** 2019-08-20

**Authors:** N. J. Rowbotham, S. J. Smith, Z. C. Elliott, P. A. Leighton, O. C. Rayner, R. Morley, A. R. Smyth

**Affiliations:** 1 Division of Child Health, Obstetrics & Gynaecology, Evidence Based Child Health Group, Obstetrics & Gynaecology, Queens Medical Centre, University of Nottingham, E Floor East Block, Nottingham, NG7 2UH UK; 2 Parent of children with CF, Nottingham, UK; 3 Faculty of Medicine & Health Sciences, University of Nottingham, Nottingham, UK; 4 Person with CF, Plymouth, UK; 5 James Lind Alliance, York, UK

**Keywords:** James Lind Alliance, Priority setting partnership, Cystic fibrosis, Videoconferencing, Patient involvement, Social media, Cross infection

## Abstract

**Plain English summary:**

Cystic fibrosis (CF) is the commonest life-limiting inherited disorder in the UK. It affects many parts of the body including the lungs and gut leading to increased infection and problems digesting food. People with CF need to undergo many treatments each day throughout their whole lives. These include tablets, inhalers and breathing exercises, which are a huge burden, taking up several hours every day

It is therefore, really important that the treatments we give are supported by good evidence, usually gathered from clinical trials. Unfortunately, we do not have good evidence for many of the CF treatments. We recently ran an exercise known as a James Lind Alliance Priority Setting Partnership (JLA PSP) to find out which the CF community feel are the top priority research questions. People with CF and those who look after them suggested questions to be answered by clinical trials. Through a series of online surveys and workshops these were then shortlisted to give a final top ten.

Due to infection risk people with CF are advised not to mix, this meant we had to do things differently to the usual way JLA PSPs are carried out. We used videoconferencing to enable multiple people with CF to participate. Surveys were accessible online and promoted through social media.

**Abstract:**

**Background**

The James Lind Alliance (JLA) method is well recognised for setting research priorities. The JLA approach involves a combination of surveys and workshop interactions between patients, carers and health care professionals to identify and agree on a “top ten” list of research questions. Respiratory infection is one of the hallmarks of cystic fibrosis (CF). To avoid cross infection, patients are advised not to meet face to face, preventing us following standard JLA methodology. Here we describe adaptations made during our recent JLA Priority Setting Partnership (PSP) in CF.

**Methods**

We elicited and prioritised research questions, using sequential online surveys, promoted through social media. People with CF participated in steering committee meetings and the final workshop, using videoconferencing. Alterations to workshop methodology enabled participants attending in person and those joining remotely, to contribute equally. We also altered the JLA methodology to include “lone” questions, asked by only one survey respondent. We are now working with the CF community to co-produce research projects that answer these top ten.

**Results**

There were 482 respondents, from 23 countries, who submitted 1080 questions. Increases in the number of responses occurred just after promotion on social media. Use of videoconferencing enabled participation of multiple people with CF and ensured participation from anywhere in the world, including hospital inpatients. Inclusion of lone questions resulted in one being included in our top ten.

**Conclusions**

There is no “one-size-fits-all” for patient involvement methodologies. Through altering the JLA methods to fit our patient group we achieved wide participation. We believe that methods used in our project may also be applied to future partnerships to increase participation, especially where people may be hospitalised or be unable to travel. The methodology we are developing through the JLA PSP CF2 project may be useful for other PSPs to follow.

## Background

Cystic Fibrosis (CF) is a life limiting inherited multi-system disorder with a high treatment burden but relatively little good quality evidence to guide treatment decisions [1]. In the UK, 10,000 people have CF [2] and 70,000 adults and children worldwide [3] are affected by the condition. This leaves a relatively small population to take part in clinical trials. It is therefore vital that the trials that do take place are those of top priority to the CF community.

The James Lind Alliance (JLA) method is well respected for setting research priorities. Their established methodology [4] involves a combination of surveys and workshop interactions between patients, carers and health care professionals to identify and agree on a “top ten” list of priorities. To date there have been over 50 Priority Setting Partnerships (PSP) across a wide field of medical conditions, conducted worldwide, although the majority have taken place within the UK [5].

Recently we undertook a JLA PSP in CF [6]. Respiratory infection is one of the hallmarks of CF and the risk of cross infection with particular bacteria that people with CF are more susceptible to, and with potentially devastating consequences, means that patients are advised never to meet face to face [7]. The US CF Foundation recommends that other approaches, such as videoconferencing and the use of online materials [8] are used to replace face-to-face contact where patient engagement is important. Here we describe how we adapted the JLA PSP process using video and online methods to allow maximal participation and involvement of patients, without putting them at risk. We believe that methods used in our project can be applied to future partnerships to increase participation. This may be especially useful in conditions where contributors may be hospitalised or may be unable to travel due to frailty or responsibilities as carers.

## Methods

A James Lind Alliance Priority Setting Partnership (JLA PSP) in CF was carried out at the University of Nottingham and commenced in March 2016. The top ten research priorities for clinical research in CF were agreed at a final workshop meeting in Jan 2017 [6]. Here we describe where we deviated from traditional JLA methodology in order to avoid cross infection and increase our reach and participation.

### JLA PSP methodology

The James Lind Alliance methodology is well defined and usually follows a pathway which includes; development of a steering group with face to face meetings; an online/paper survey to gather uncertainties; organization of responses and checking against current evidence; interim priority setting to shorten the list of questions; a final workshop with a mixture of small and whole group discussions where all participants are present in person. The workshop brings together representatives from both the professional and lay communities who jointly refine the final top ten priorities.

**Fig. 1:**
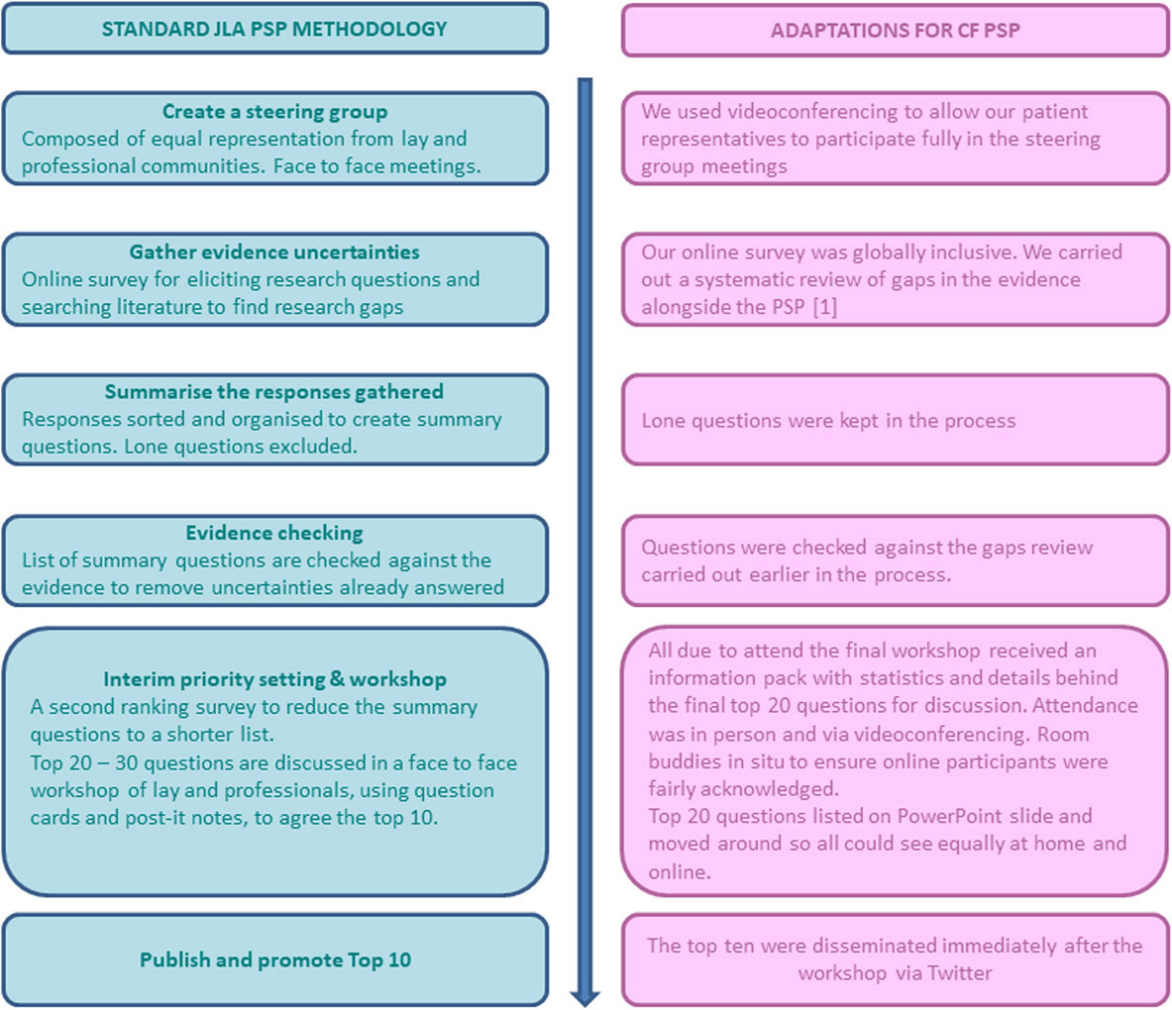
Standard JLA methodology and adaptions made in the CF PSP. Standard JLA methodology is shown on the left hand side, adaptions for the CF PSP shown on the right hand side

Below we outline where our methodology deviated from the traditional methods and this is also represented in Fig. 1.

### Use of online surveys

We used two online surveys:

- An elicitation survey to collect research questions from the patient and clinical community
- A prioritisation survey, where the patient and clinical communities ranked the final questions in order of importance.

**Fig. 2:**
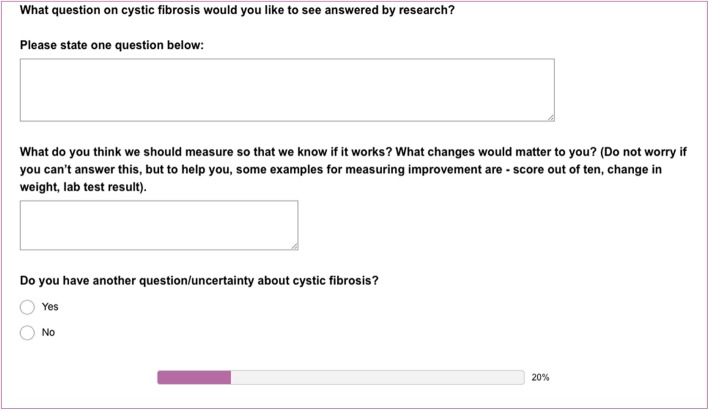
Elicitation survey. This screen shot shows how research questions were collected. A free text box was used for people to leave their answer in a narrative or story as well as by simple question format

These surveys were designed to be completed easily on a mobile device (e.g. smart phone). They collected a minimum of demographic information to ensure they could be completed in a short time. The elicitation survey allowed up to five free text research questions to be submitted, with a suggested outcome measure for each (Fig. 2). The prioritisation survey used a “drag and drop” interface to allow the participant to compile and rank their top ten research questions.

### Social media promotion

A bespoke Twitter account was set up @questionCF with the associated hashtag #questionCF. This was managed by members of the steering group and aimed to promote the online surveys and increase participation.

### Videoconferencing

**Fig. 3:**
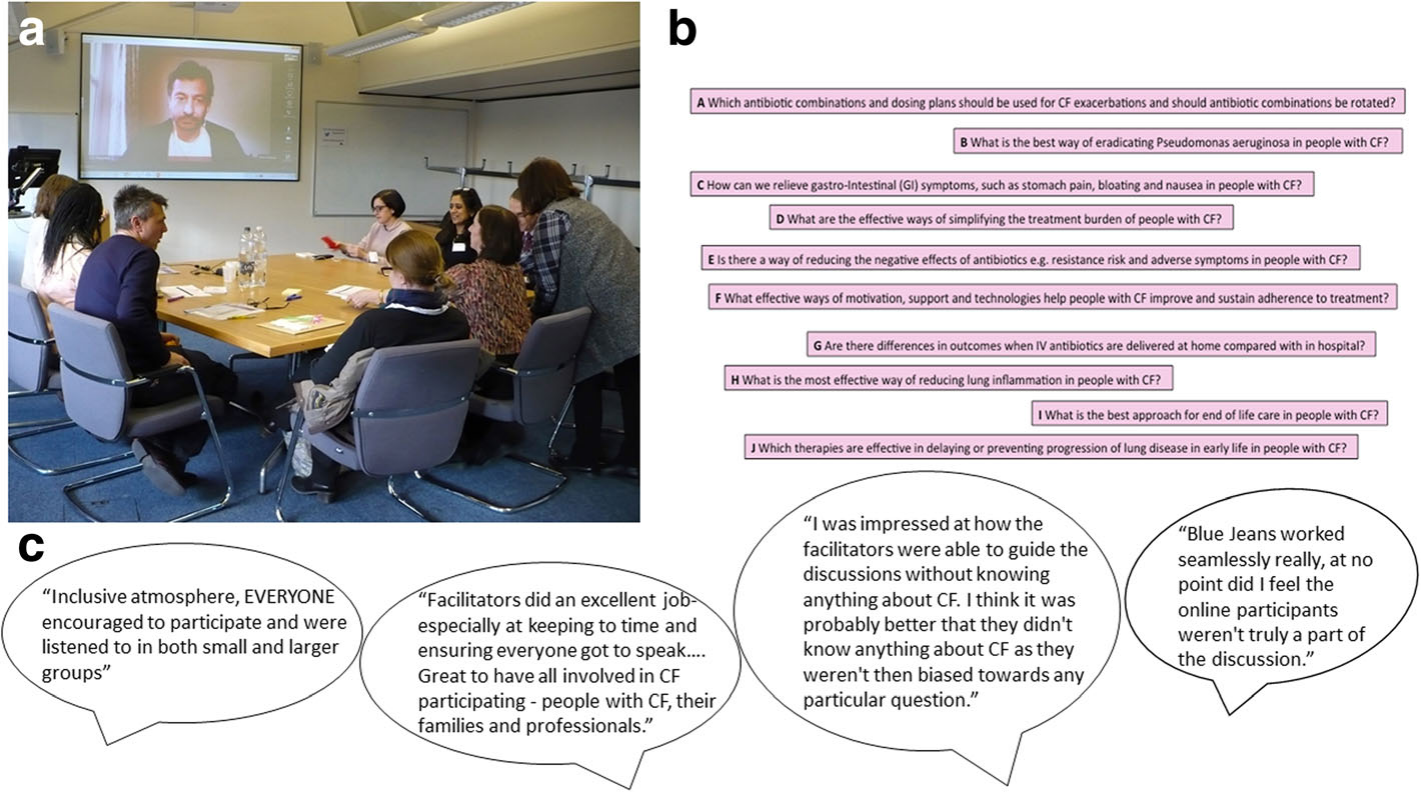
Remote participation during the final workshop. **a** Photograph showing room set up for videoconferencing via the BlueJeans^TM^ platform. **b** Example of electronic question list. This was made in PowerPoint and sharable to each screen. Each question could be moved as it was discussed with participants. **c** Quotes from feedback from remote participants

To prevent risk of spread of infection, people with CF are advised not to meet face to face with others who have the condition [8]. This presented us with a unique challenge as the JLA Guidebook describes steering group meetings and the final workshop occurring face to face. We used online video conferencing methods through the BlueJeans™ platform to avoid cross infection and widen participation (Fig. 3a.). These were used both for steering committee meetings and for the final workshop.

### Final workshop adaptations

Prior to the workshop, test calls were carried out with all those participants planning on joining remotely. This allowed them to gain familiarity with the software and any necessary trouble shooting to be undertaken. Basic etiquette rules to facilitate the workshop such as muting of microphones when not speaking to avoid background noise and not having light source behind the participant were also covered.

To ensure that those joining the final workshop remotely had their voice heard, a non-participating “room-buddy” was present in person at the workshop. The “room buddy” was a steering group member with the specific task of advocacy for one remote participant.

Usually in JLA PSP workshops question cards are moved around on tables to help the group decide on rankings [4, 9]. The steering group decided this was not a practical approach for our final workshop, as remote participants were unable to see what was taking place in the room. To give everyone an equal view of the questions and their current ranking, an online real-time visual aid of the questions was used and controlled by a “list-master” in each room (Fig. 3b). This visual aid was shared with the remote participants so that all participants had an equal view of how the rankings were changing. The list-master only moved a question when consensus was reached in the small groups. All participants, whether attending in person or remotely were sent an information pack prior to the workshop including question cards (with details of the candidate questions, numbers of ‘votes’ and breakdown of lay versus profession votes) to make it more engaging and easier for participants to play their part.

At the end of the process a short survey was sent out to remote participants to find out about their experience of participating via videoconferencing.

### Lone questions

Through the elicitation survey, we asked participants to tell us what they thought were the most important questions that needed answering by clinical research. We set aside any questions which did not relate to treatment uncertainties and those which had already been answered. Prior to commencing this analysis we undertook a systematic review of gaps in the evidence for treatment decisions in CF which helped with this process [1]. We sorted and grouped the resulting questions to produce a shorter list of questions which could go through to the ranking survey. The final list of questions to go to the prioritisation survey was selected using a modified Delphi technique [10], conducted amongst all members of the steering committee. Many PSPs discard questions which were only suggested by one respondent at this stage [11, 12]. We did not discard questions simply because they were suggested by one respondent only, as we felt that every question deserved a chance of progressing.

### Dissemination of final top ten

The final top ten questions were shared with the patient and clinical community immediately after the final workshop, via our dedicated Twitter account (@questionCF). We felt it was important to release the top ten immediately so that those who had contributed would find out straight away. We produced a press release and key stakeholders, such as the CF trust and NIHR, were updated. Postcards with the Top ten were produced and shared by steering group members with the patient community and with their clinical colleagues. Several discussions were held with the National Institute for Health Research (NIHR) to discuss taking ideas from the top 20 questions forward and funding opportunities for them.

## Results

Through the use of online survey accessible to all, we reached 2.5% of the UK’s CF population and 0.45% of the world’s CF population [6].

**Fig. 4:**
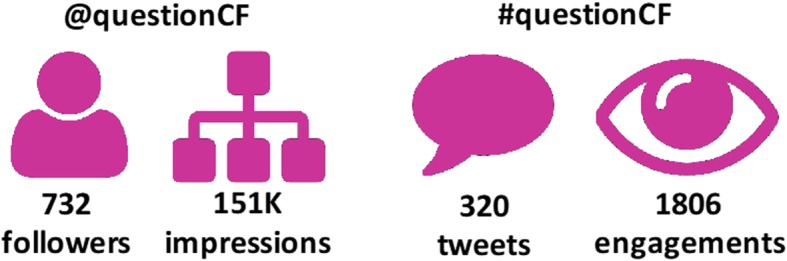
Twitter analytics for the @QuestionCF account during the course of the priority setting partnership. Impressions are the total number of views of a conversation and engagements are the number of interactions people have with our content

Over the course of the PSP our @questionCF twitter account gained 732 followers with 151,000 impressions (total number of views of a conversation). Our hashtag #questionCF was used 320 times with 1806 engagements (the number of interactions people have with our content) (Fig. 4). Over half of our followers were UK based but we have had interactions with accounts from North America (32%), Australia (4%), the rest of Europe (6%) and the Middle East (2%). Our twitter presence continues to grow with a current count (April 2019) of 1160 followers. Through twitter conversations with US counterparts we forged a collaboration with researchers from the CF Foundation.

The use of videoconferencing at steering group meetings allowed two people with CF to participate in each meeting. Had we not used this method, only one person with CF could have attended the meeting in person. Our steering group members were drawn from all over the UK and videoconferencing allowed colleagues to take part who could not travel, or who had conflicting clinical commitments. Six people attended the final workshop remotely, including one who was a hospital inpatient and an observer from the Cystic Fibrosis Foundation in the USA.

We evaluated participants’ experience of joining the workshop remotely and five of the six remote participants completed feedback; three said overall they were “very satisfied” with the experience and two “satisfied”. Quotes from feedback from the remote participants are shown in Fig. 3c. Having test calls prior to the event were felt to be very beneficial. Areas for improvement included ensuring the setup of the room was as inclusive as possible and using roaming microphones in group discussions to make sure remote participants could hear clearly.

**Fig. 5:**
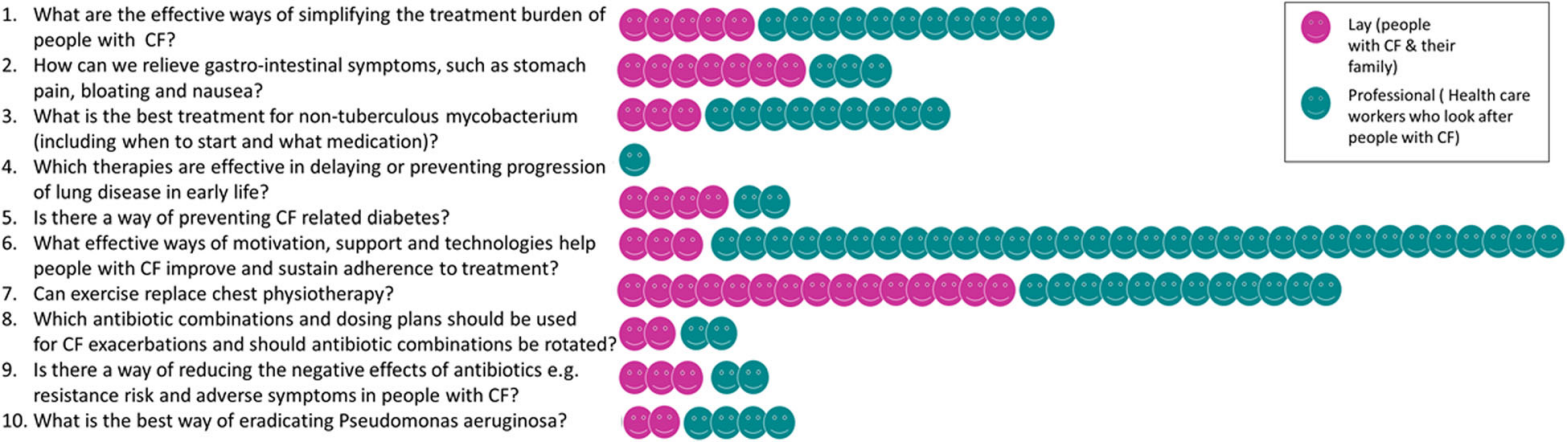
Breakdown of how many individual questions fed into the final top 10 questions. Each individual face represents one person submitting a question, pink represents people with CF and their family and friends, green represents health care professionals

From a total of 39 questions classified as “lone questions”, six were carried forward to the public ranking survey. One of these was in the final top ten at position number four *“Which therapies are effective in delaying or preventing progression of lung disease in early life in people with Cystic Fibrosis?”*. Figure 5 shows the breakdown of how many individuals submitted the questions that fed into the final top ten.

Our immediate dissemination of the Top 10 via Twitter™ proved very popular with the tweet of the Top 10 questions gaining a potential reach of 59,061 people from the many retweets and commenting on the post. From the press release several online news outlets picked up the story and the CF trust produced a short video on the top ten. The postcards we produced have been very useful in providing interested parties with a “take home” visual guide to the Top 10.

We are pleased that following on from our discussions with the NIHR, a Health Technology Assessment (HTA) call was released in April 2018, with seven of the eight areas with calls for funding related to our JLA top ten. A further call to apply for NIHR funding to support clinical research in CF, with a particular focus on the JLA top 10, is planned.

## Discussion

The importance of cross infection in CF has meant that our PSP had to adapt the standard approach, described in the JLA Handbook [4]. The use of online videoconferencing avoided the risk of cross infection; allowed participation by those who could not travel (due to hospitalisation, distance or family responsibilities); and minimized participation time. The inclusion of remote participants throughout the process worked well and feedback indicated that they were still able to participate fully.

Whilst some of the issues we encountered were unique to CF, there are many other instances where our adapted methodology may enhance inclusivity. The Digital Technology for Mental Health PSP used an online Twitter™ chat to gather uncertainties amongst a population where an online survey would be difficult for some participants [13]. Other PSPs have used a combination of online surveys, paper surveys, telephone conversations, focus groups and face to face questioning at public events to gather their uncertainties [14, 15]. Our PSP is the first to use remote participation (C Whiting JLA NIHR personal communication). Many conditions and life commitments make it more difficult to travel to participate in a face to face workshop and the use of a virtual workshop may overcome this. The traditional methodology of a face to face workshop may exclude people who feel uncomfortable in group situations and remote participation, from familiar surroundings, may alleviate this problem. This allows engagement to be more balanced and not restricted to a few vocal individuals.

In the planning and delivery of our workshop, we aimed to ensure equality amongst the contributors to allow everyone to have an equal say, regardless of whether they were in the room or joining remotely. Future PSPs may be able to take this process further and run virtual workshops where all participants join remotely which may achieve greater equality. In addition to widening access to participation, there could be other benefits such as cost savings and reduced need for travel. However it is also likely that PSP engagement through online surveys and social media may not be comfortable for all potential participants (e.g. the elderly). This may warrant a well-designed study to compare the standard with an adapted methodology to compare the two.

The demographic distribution of the CF population means that most are familiar with social media and it is a safe and popular way to interact with other people with CF without fear of cross infection [16, 17]. Our use of Twitter™ during this project increased our reach within the CF community and allowed global participation and collaboration. The number of people who interacted with our questionCF posts showed that the CF community are engaged in and passionate about research.

Many PSPs in other areas have discarded questions where they are suggested by one respondent alone. When we summarised our submitted questions, we kept lone questions in a separate list. We decided to allow these “lone” questions to progress by adding them to the list of summarised questions. One of these remained in the process all the way to the final top ten. This would have been lost from the top ten if we had followed the practice of earlier PSPs. It highlights the importance of the steering group in guiding the refining of questions and ensuring the process remains true to what the CF community were telling us.

The NIHR plans to invite funding applications for CF research based on our JLA top ten. We feel that co-production of CF research should continue beyond setting research priorities. Some of the top ten questions are still very broad, for example: “What are the effective ways of simplifying the treatment burden of people with Cystic Fibrosis?” To develop this question into a testable hypothesis we need to understand:

- What are the most burdensome aspects of treatment for people with CF?
- Does this vary with age, sex, and employment or education status?
- Would it be acceptable to the person with CF to have a reduction in treatment burden at the expense of a more rapid decline in lung function?

We are currently undertaking a project to explore a number of the top ten questions in greater depth in partnership with the CF community, using a combination of online surveys and focus groups (known as JLA PSP CF2). Our goal is to formulate testable hypotheses for clinical research studies. Our protocol for this is published on the University of Nottingham Research Repository [18].

Although the focus of the JLA is to further the evidence base through clinical trials of treatment interventions, other research methodologies, such as registry studies, may also help resolve clinical uncertainties in CF. In the US, this approach has been used by the US CF Foundation, through the “Insight CF” survey [19]. This encouraged the patient community to provide questions which could be answered through their registry.

## Conclusions

There is no “one-size-fits-all” for patient involvement methodologies. Through altering the JLA methods to fit our patient group we increased participation. We believe that methods used in our project may also be applied to future partnerships to increase participation, especially in conditions where people may be hospitalized, may be unable or too busy to travel. The methodology we are developing through JLA PSP CF2 / QuestionCF2 project will be useful for other PSPs to follow to help turn their top ten questions into fundable research projects.

## Data Availability

Anonymised data from the PSP process are available from the corresponding author on reasonable request.

## Abbreviations

CF: Cystic fibrosis
HTA: Health Technology Assessment
JLA: James Lind Alliance
NIHR: National Institute for Health Research
PSP: Priority Setting Partnership
